# A Virulence Essential CRN Effector of *Phytophthora capsici* Suppresses Host Defense and Induces Cell Death in Plant Nucleus

**DOI:** 10.1371/journal.pone.0127965

**Published:** 2015-05-26

**Authors:** Joseph Juma Mafurah, Huifei Ma, Meixiang Zhang, Jing Xu, Feng He, Tingyue Ye, Danyu Shen, Yanyu Chen, Nasir Ahmed Rajput, Daolong Dou

**Affiliations:** Department of Plant Pathology, Nanjing Agricultural University, Nanjing, 210095, China; Rutgers University, UNITED STATES

## Abstract

*Phytophthora capsici* is a soil-borne plant pathogen with a wide range of hosts. The pathogen secretes a large array of effectors during infection of host plants, including Crinkler (CRN) effectors. However, it remains largely unknown on the roles of these effectors in virulence especially in *P*. *capsici*. In this study, we identified a cell death-inducing CRN effector PcCRN4 using agroinfiltration approach. Transient expression of *PcCRN4* gene induced cell death in *N*. *benthamiana*, *N*. *tabacum* and *Solanum lycopersicum*. Overexpression of the gene in *N*. *benthamiana* enhanced susceptibility to *P*. *capsici*. Subcellular localization results showed that PcCRN4 localized to the plant nucleus, and the localization was required for both of its cell death-inducing activity and virulent function. Silencing *PcCRN4* gene in *P*. *capsici* significantly reduced pathogen virulence. The expression of the pathogenesis-related gene *PR1b* in *N*. *benthamiana* was significantly induced when plants were inoculated with *PcCRN4*-silenced *P*. *capsici* transformant compared to the wilt-type. Callose deposits were also abundant at sites inoculated with *PcCRN4*-silenced transformant, indicating that silencing of *PcCRN4* in *P*. *capsici* reduced the ability of the pathogen to suppress plant defenses. Transcriptions of cell death-related genes were affected when *PcCRN4*-silenced line were inoculated on *Arabidopsis thaliana*, suggesting that PcCRN4 may induce cell death by manipulating cell death-related genes. Overall, our results demonstrate that PcCRN4 is a virulence essential effector and it needs target to the plant nucleus to suppress plant immune responses.

## Introduction

During plant-pathogen interactions, pathogens secrete an array of effector proteins to aid infection and establishment of parasitic lifestyles by modulating host cell defenses [1–3]. At the same time plants defend themselves through several mechanisms of local immune system of each cell and systemic acquired resistance (SAR) of the whole plant [1,2]. The plant immune system and its interplay with pathogen effectors are widely illustrated as a four-stage zigzag model. When plants are attacked by microbes, they recognize microbe or pathogen-associated molecular patterns (PAMPS) which elicits PAMP-triggered immunity (PTI). Effective microbes have evolved a huge and diverse effector repertoires to counter PTI by suppression and subsequently enhance susceptibility (Effector-triggered susceptibility, ETS) [1,4]. A dynamic interaction between host immune responses and pathogen effectors has been widely reported in different pathosystems [5,6]; and identifying pathogen effectors and discovering their functions have become as essential routes to understand pathogen establishment.

*Phytophthora* pathogens encode a large number of effectors to interfere with host plant cell physiology and function. They belong to the fungus-like oomycetes, which are evolutionarily closely to algae in the kingdom Stramenopila [7,8]. *Phytophthora* species are arguably the most destructive pathogens of many dicotyledonous plants and contain many well-known pathogens including *P*. *capsici* [9,10]. *P*. *capsici* is a broad-host-range pathogen, and may cause damage to many economically important vegetables including all cucurbits, pepper, tomato and eggplant. The disease occurrence and acuteness have significantly increased in recent decades worldwide [10]. However, its pathogenesis mechanisms are still largely unknown. Similar to other well-studied *Phytophthora* pathogens, such as *P*. *infestans* and *P*. *sojae*, *P*. *capsici* also encodes a large number of host cytoplasmic effectors, including 357 putative RXLR (Arg-any amino acid-Leu-Arg) and 84 CRN (Crinkler, Crinkling and necrosis inducing protein) effectors [11]. The RXLR effectors contain a conserved N-terminal motif that is involved in translocation inside host cells [12,13] and C-terminal domains that may manipulate plant immunity responses [5,14].

CRN effectors are another group of pathogen proteins presumed to enter the host cytoplasm [15,16]. Interestingly, CRN effectors are also modular proteins. Their N-termini contain the predicted signal peptide and a conserved motif, FLAK (F, Phe; L, Leu; A, Ala; and K, Lys), which is essential for effector translocation to host cells [17]. The C-terminal effector domains have various functions, such as inducing cell death (CD) and suppressing host immunity [15,18–20]. They usually target host cell nucleus to efficiently exert their biological functions [17,20]. Analyses of three necrosis-inducing CRN effector domains (DN17, D2, and DXZ) revealed differences in the timing and occurrence of cell death in *N*. *benthamiana* [21]. Several CRN effectors studied so far have shown that they enhance pathogen virulence. For example, CRN8 with a functional RD kinase enhanced virulence of *P*. *infestans* when it was expressed *in planta*. In contrast, its dominant-negative CRN8^R469A;D470A^ resulted in reduced virulence [22]. Liu *et al*. have demonstrated that silencing of *PsCRN63* and *PsCRN115* jointly in *P*. *sojae* led to a reduction of virulence on soybean [18]. In a recent study, *N*. *benthamiana* expressing *PsCRN70* gene increased its vulnerability to *P*. *parasitica* [23]. However, the virulence mechanisms of *P*. *capsici* CRN effectors are mostly unknown.

In this report, a *P*. *capsici* CRN effector PcCRN4 was identified by cell death-inducing assay *in planta*. It can induce CD on the tested plants and the encoding gene was dramatically induced at the infectious stages. Silencing the genes in a stable transformant leads to a significant reduction of virulence on plants and its transient expression *in planta* enhances plant susceptibility. Furthermore, ROS accumulation, callose deposition and expressions of *PR1b* and CD-related genes were altered in plants inoculated with the *PcCRN4*-silenced transformant. Collectively, we propose a role for CRN effectors in *P*. *capsici* virulence.

## Materials and Methods

### Plasmid construction

The oligonucleotides used for the following plasmid constructs are documented in S1 Table. For PVX recombinant constructs, 47 genes encoding CRN effectors were amplified and inserted into the modified PVX vector [6] using the *Sma* I and *Not* I restriction sites. For GFP fusion constructs, *PcCRN4*, *PcCRN4*:*NES* and *PcCRN4*:*nes* were amplified using combinations of oligonucleotide primers PcCRN4-F and PcCRN4-R, PcCRN4-F and PcCRN4-NES-R, and PcCRN4-F and CRN4-nes-R, respectively. PCR products were digested with *Sma* I and *Xba* I restriction enzymes, and then inserted into the expression vector pBinGFP2 [24]. To make the construct for gene silencing in *P*. *capsici*, partial sequence of *PcCRN4* was amplified using PrimeSTAR HS DNA Polymerase (Takara code DR010A) and subsequently inserted into pTOR [13,25]. All the generated plasmids were validated by sequencing by GenScript Corporation Company (Nanjing, China).

### *Agrobacterium tumefaciens* infiltration assays

The *A*. *tumefaciens* infiltration assays were performed as previously described methods [26], except that an *A*. *tumefaciens* strain GV3101 [27] was used. For infiltration, each recombinant strain was cultured in Luria-Bertani broth supplemented with 50 μg ml^-1^ kanamycin at 28°C to 30°C and 220 rpm for 48 h. The bacterial cells were collected by centrifugation (3,000g, 5 min), washed three times with 10 mM MgCl_2_, and then resuspended in 10 mM MgCl_2_ to an optical density at 600 nm of 0.4. Infiltrations were performed on 6–8 week-old *Nicotiana benthamiana*, *N*. *tabacum* and *Solanum lycopersicum* plants. Plants were grown and maintained all through the experiments in a greenhouse with favorable temperature of 22–25°C and high light intensity under a 16-h/8-h light/dark photoperiod. For CD triggering assays, *A*. *tumefaciens* cells loaded with the respective constructs were infiltrated into the leaves by pressure infiltration by placing a small nick on each leaf with a needle, then 30–50 ml of cell suspension was infiltrated through the nick using a syringe without a needle. Symptoms were monitored from 4 to 8 days after infiltration, and photographs were taken after 5 days for *N*. *benthamiana*, 8 days for *N*. *tabacum* and 15 days for *S*. *lycopersicum*. These experiments were repeated at least three times.

### Pathogenicity assay

*Phytophthora capsici* infection assays were performed by droplet inoculations of zoospore solutions of the *P*. *capsici* isolate LT263 (10 x 100 of zoospores μl^-1^) on detached *N*. *benthamiana* and *Arabidopsis thaliana* leaves. At least ten independent *N*. *benthamiana* leaves (4 weeks old) and *A*. *thaliana* leaves (6–8 weeks old) were tested per construct combination. *Agrobacteria* cells containing *GFP*:*PcCRN4*, *PcCRN4*:*NES*, *PcCRN4*:*nes* or *GFP* (control) were infiltrated into *N*. *benthamiana* leaves, and 24 h later the leaves were detached and inoculated with *P*. *capsici* zoospores on the abaxial side. Diseased plant tissues (36 hpi) were stained by Trypan blue as described methods [28]. The diameters of the diseased lesions were measured at 24, 36 and 48 hpi, and photographed at 36 hpi. The assay was repeated at least three times. Dunnett’s test was used for statistical analysis.

### *Phytophthora capsici* inoculation and *in vitro* samples

*Phytophthora capsici* wild-type strain LT263 was grown in petri dishes on V8 agar medium in a dark climate chamber at 25°C for 4 days and under standard light at 22°C for 3 days. To induce zoospore release, the cultures were washed twice with sterilized distilled water and released in 10 ml of sterile distilled water per plate by placing at 4°C for 0.5 h followed by incubation at 25°C for about 1 h. Release of zoospores was monitored; their numbers counted with a hemocytometer under a microscope, and adjusted to 100 zoospores μl^-1^. The detached leaves were inoculated with 20 μl droplets of the zoospore solution and samples were collected after 3 h, 6 h, 12 h, 24 h, 36 h and 48 h then frozen in liquid nitrogen and stored at -80°C. In addition to samples to be taken during the infectious stages, zoospores (ZO), germinating cysts (GC), and mycelia (MY) grown *in vitro* were also prepared. ZO were collected in 50 ml centrifuge tube, shaken vigorously, then observed under the microscope. For GC, an equal volume of V8 broth was added to the zoospore suspension, mixed and allowed to stand for 1 h at 25°C. The mixture was centrifuged at 2000 rpm for 5 minutes and pellets collected. The mycelia were grown in 1 ml pea broth, infected with 20 μl of inoculum at 22°C, and harvested 48 hpi by collecting the mycelial mat into 10 ml tubes. The samples were placed in the controlled incubator with the same conditions as the leaf samples, and then harvested with 5 minutes centrifugation at 1,200 g. The pellets were collected and frozen in liquid nitrogen after the supernatant was removed.

### RNA extraction and real time quantitative RT-PCR

Total RNA was isolated from frozen leaf tissue (RNAsimple Total RNA kit, Tiangen) according to the manufacture’s protocols. Its quality was confirmed by agarose gel electrophoresis, then the quantity was measured with a spectrophotometer (Nanodrop ND-1000). The total RNA was treated with DNase to remove genomic DNA contamination and cDNA was synthesized using 1000 ng of total RNA using a commercial kit (PrimeScript reagent Kit, TaKaRa) following the recommended instructions.

All the specific primers for quantitative RT-PCR (qRT-PCR) were designed for each gene (S1 Table). The experiments of qRT-PCR were performed in 20 μl reactions, including 20 ng cDNA, 0.2 μM gene-specific primers, 0.4 ul ROX Reference Dye, 10 μl of SYBR Premix ExTaq (TaKaRa), and 6.8 μl of deionized water. An ABI Prism 7500 Fast Real-Time PCR System (Applied Biosystems) was used under the following conditions: 95°C for 30 s and 40 cycles at 95°C for 5 s, 60°C for 34 s, followed by a dissociation step of 95°C for 15 s, 60°C for 1 min, and 95°C for 15 s. The relative expression levels of the tested genes were normalized to the *P*. *capsici Actin* gene.

### Protein extraction and Western blot

The experiments were performed as previously described methods [24]. Briefly, the *N*. *benthamiana* leaves were infiltrated with GFP:PcCRN4, GFP:PcCRN4:NES, GFP:PcCRN4:nes and GFP harvested at 2 dpi. Protein extractions were done using GTEN buffer (10% Glycerol, 25 mM Tris, 1 mM EDTA, 150 mM NaCl) that is supplemented with 2% PVP, 10 mM DTT and 1X Complete protease inhibitor cocktail (Roche). Samples were separated on 12% SDS PAGE gels and then transferred to PVDF membranes. The membranes were blocked for 30 minutes with 5% milk in PBS-T (0.1% tween) and probed with GFP antibody (Genscript). The treated membranes were washed 3 times in PBS-T for 5 minutes and then incubated in PBS-T with a goat anti-mouse IRDye 800CW (Li-Cor) for 40 min. After three times wash with PBS-T, the membranes were visualized using a LI-COR Odyssey scanner with excitation 700 and 800 nm to obtain images.

### Confocal microscopy

All localization studies were done on *N*. *benthamiana* leaves that were infiltrated with GFP-tagged PcCRN4, PcCRN4:NES and CRN4:nes two days earlier. The leaves were infiltrated with water to maintain cell structure after detachment and then mounted on a microscope slide. The controls were leaves infiltrated with GFP. Leaf samples were imaged using a Zeiss 710 CLSM with the excitation wavelength at 488 nm.

### Stable silencing of *PcCRN4* in *P*. *capsici*

A partial sense sequence of *PcCRN4* was cloned into the pTOR vector and transformed into wild-type isolate LT263 as described previously [26]. The transformants were maintained on V8 agar plates supplemented with 50 μg ml^-1^ of G418 and gene expression levels from the mycelium were determined by qRT-PCR as described previously.

### Oxygen burst detection and callose deposition assay

Oxygen burst was observed based on H_2_O_2_ accumulation after staining *N*. *benthamiana* leaves with diaminobenzidine (DAB) [28]. Briefly, the leaves were infiltrated with *PcCRN4* constructs adjusted to OD 0.2 then 24 hpi, they were inoculated with *P*. *capsici* zoospores (10 x 100 zoospores μl^-1^). The leaves were then soaked in DAB at 1 mg ml^-1^ 12 hpi thereafter maintained at 25°C for 8 h. Leaf sections were cleared by boiling in 95% ethanol for 15 min and incubated in bleaching solution until all the chlorophyll was completely bleached. The experiments were repeated at least three times.

Callose deposition assays for *Arabidopsis* were modified as follows from previously described procedures [29]. Briefly, leaves of 8-week-old *Arabidopsis* ecotype Columbia-0 plants, were inoculated with 3-day-old *P*. *capsici* mycelium (5 mm). After 6 and 24 h, leaf disks from inoculated areas were excised with a cork borer from *Arabidopsis* (5 mm in diameter), and then incubated in bleaching solution of 1:1:1:1:8 (phenol: glycerol: lactic acid: water: ethanol) at 60°C until the leaf disks were cleared of chlorophyll. The cleared leaf disks were washed three times with distilled water, and then immersed in 0.01% aniline blue in 150 mM K_2_HPO4 (pH 9.5) and incubated in the dark for 4 hours. The stained leaf disks were mounted with 60% glycerol on glass slides and observed from the adaxial surface of the disk by epifluorescence microscopy using ultraviolet light.

## Results

### Identification of *PcCRN4* homologs and cell death inducing activities in three plants

**Fig 1:**
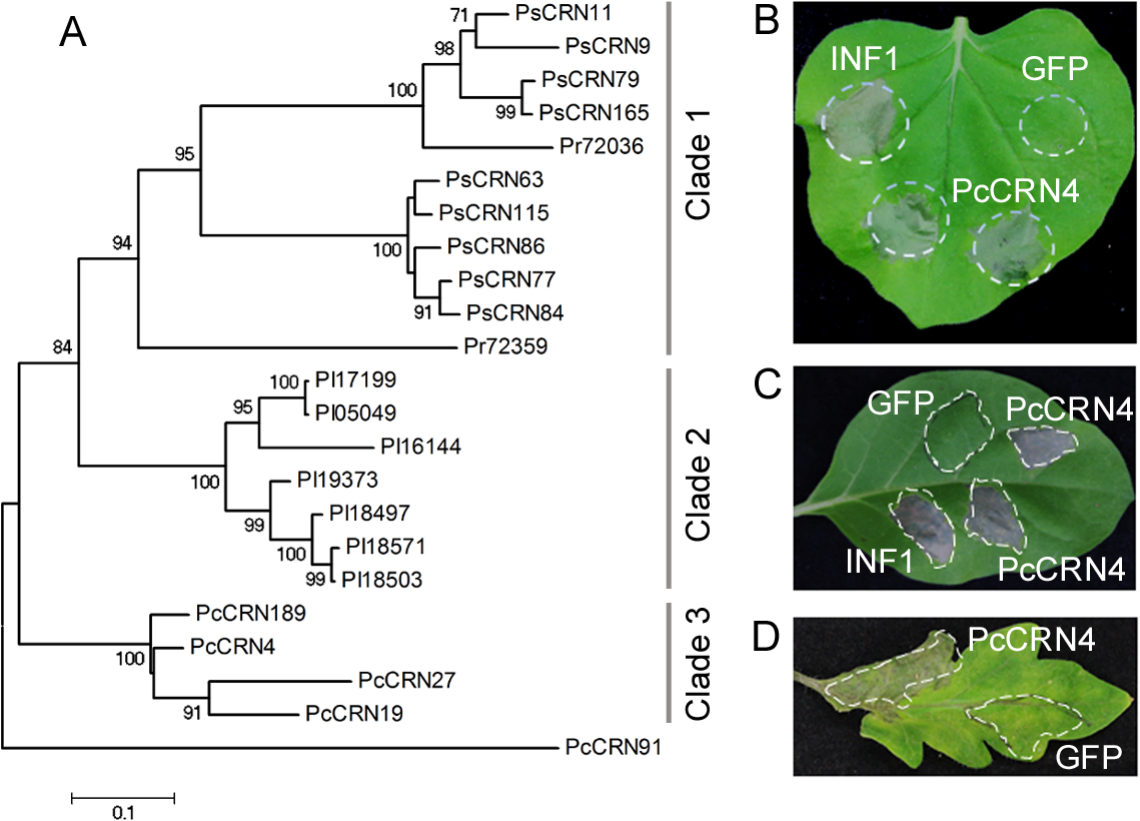
Identification of PcCRN4 orthologs and cell death assay of PcCRN4. (A) Phylogenetic relationships of PcCRN4 orthologs in four *Phytophthora* species. The phylogenetic tree was constructed with amino acid sequences using MEGA 5 with the neighbor-joining method, 1,000 replicates, and pairwise-deletion option. CRN genes, designated Ps, Pi, Pr and Pc correspond to *P*. *sojae*, *P*. *infestans*, *P*. *ramorum* and *P*. *capsici* respectively. PcCRN4 induces cell death in *Nicotiana benthamiana* (B), *N*. *tabacum* (C) and *Solanum lycopersicum* (D). The leaves were infiltrated with *Agrobacterium tumefaciens* carrying GFP:PcCRN4 and the indicated controls. The photographs were taken 5 days (B), 8 days (C) and 15 days post infiltration (D), respectively. All the experiments were repeated at least three times.

To investigate the roles of CRN effectors from *P*. *capsici*, we screened CRN effectors that can trigger cell death (CD) using the potato virus X (PVX)-mediated transient expression in *N*. *benthamiana* and *N*. *tabacum* [30]. In total 47 *PcCRN* genes (S2 Table), three genes (*PcCRN4*, *PcCRN23* and *PcCRN42*) exhibit CD-inducing activities in *N*. *benthamiana* and *PcCRN4* induced CD in both plants while no CD activities were observed in all other genes (S1 Fig). The *PcCRN4* gene has been reported as PcCRN83_152 and induced CD in *N*. *benthamiana* [20]. We named the genes depending on their relative expression levels in *P*. *capsici* hyphae, determined by RNA-seq analysis (S2 Table), implying that *PcCRN4* was the 4^th^ highest in terms of relative expression among the 47 *PcCRN* effectors. We selected *PcCRN4* gene for further analysis based on the following reasons. It exhibited strong CD in the tested plants at 4 dpi (days post infiltration) while the other two genes (*PcCRN23* and *PcCRN42*) induced weak cell death in *N*. *benthamiana* only at 5 dpi (S1 Fig). On the phylogenetic tree, the 22 CRNs were categorized into three clades according to origin (Fig 1A). Clade 1 include 9 members from *P*. *sojae* and 2 from *P*. *rumorum*. All 7 *P*. *infestans* genes belong to Clade 2 while clade 3 contains 4 members from *P*. *capsici*, suggestive of a specie-specific expansion of this orthologous gene group. Its homologs PsCRN63 from *P*. *sojae* and PiCRN2 from *P*. *infestans* have been shown to elicit cell death in *N*. *benthamiana* [16,18].

Furthermore, we confirmed *PcCRN4* CD-inducing activities using plant infiltration experiments by *Agrobacterium tumefaciens*-mediated transient expression (*GFP* gene fusion with the mature *PcCRN4* gene without the predicted signal peptides) in *N*. *benthamiana*, *N*. *tabacum* and *S*. *lycopersicum*. A known elicitor of cell death, the *P*. *infestans* INF1 [31] and GFP were used as a positive and negative controls respectively. Strong necrosis phenotype was observed in *N*. *benthamiana* at 5 dpi (Fig 1B), *N*. *tabacum* at 8 dpi (Fig 1C) and *S*. *lycopersicum* at 15 dpi (Fig 1D). The results were similar for each repeat and demonstrated that PcCRN4 can trigger cell death in a variety of the tested plants.

### *PcCRN4* is expressed at infection stages

**Fig 2:**
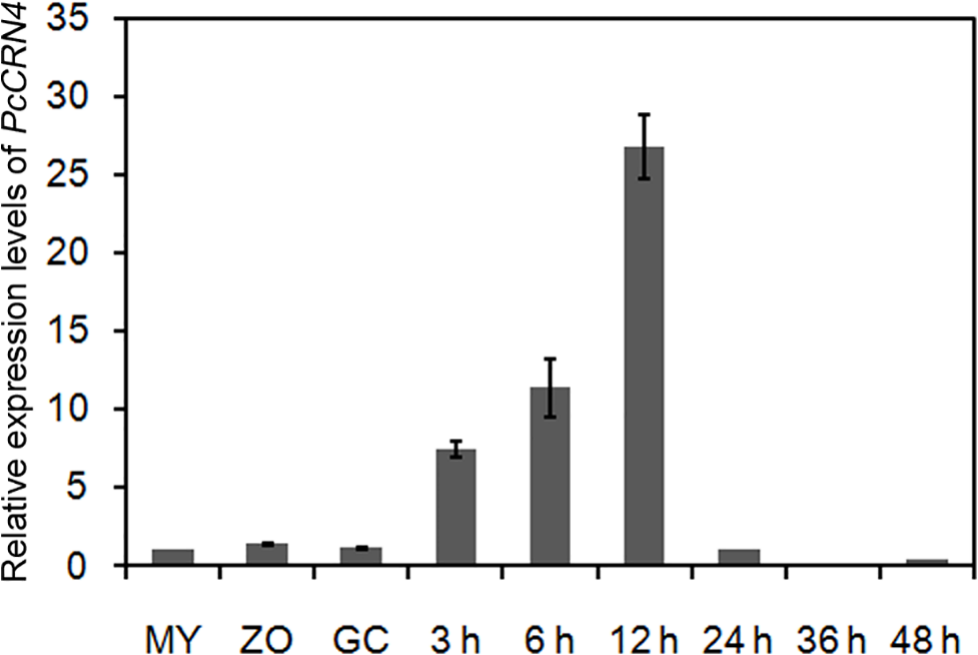
Expression profile of the *PcCRN4* gene. Relative *PcCRN4* mRNA levels were quantified by quantitative RT-PCR in samples corresponding to mycelium (MY), swimming zoospores (ZO), germinating cysts (GC) and *Nicotiana benthamiana* leaves inoculated with *P*. *capsici* zoospores at different time-points post infection. *P*. *capsici actin* transcripts were used as a reference and then normalized to the MY.

Nextly, we determined its expression levels at different stages using quantitative RT-PCR on *N*. *benthamiana* leaves inoculated with motile zoospores of *P*. *capsici*. *PcCRN4* transcripts were detectable in the mycelium (MY), zoospores (ZO) and germinating cysts (GC) at low levels; and increased during the early biotrophic phase (3 hours post-inoculation; hpi) and reached the peak at 12 hpi (Fig 2). The amount of *PcCRN4* transcripts declined in late biotrophy (24 hpi) and became barely detectable during the necrotrophic phase of the infection (36 and 48 hpi). These results suggested that the *PcCRN4* transcripts accumulate at the early stages of infection.

### PcCRN4 requires nuclear localization to trigger cell death

**Fig 3:**
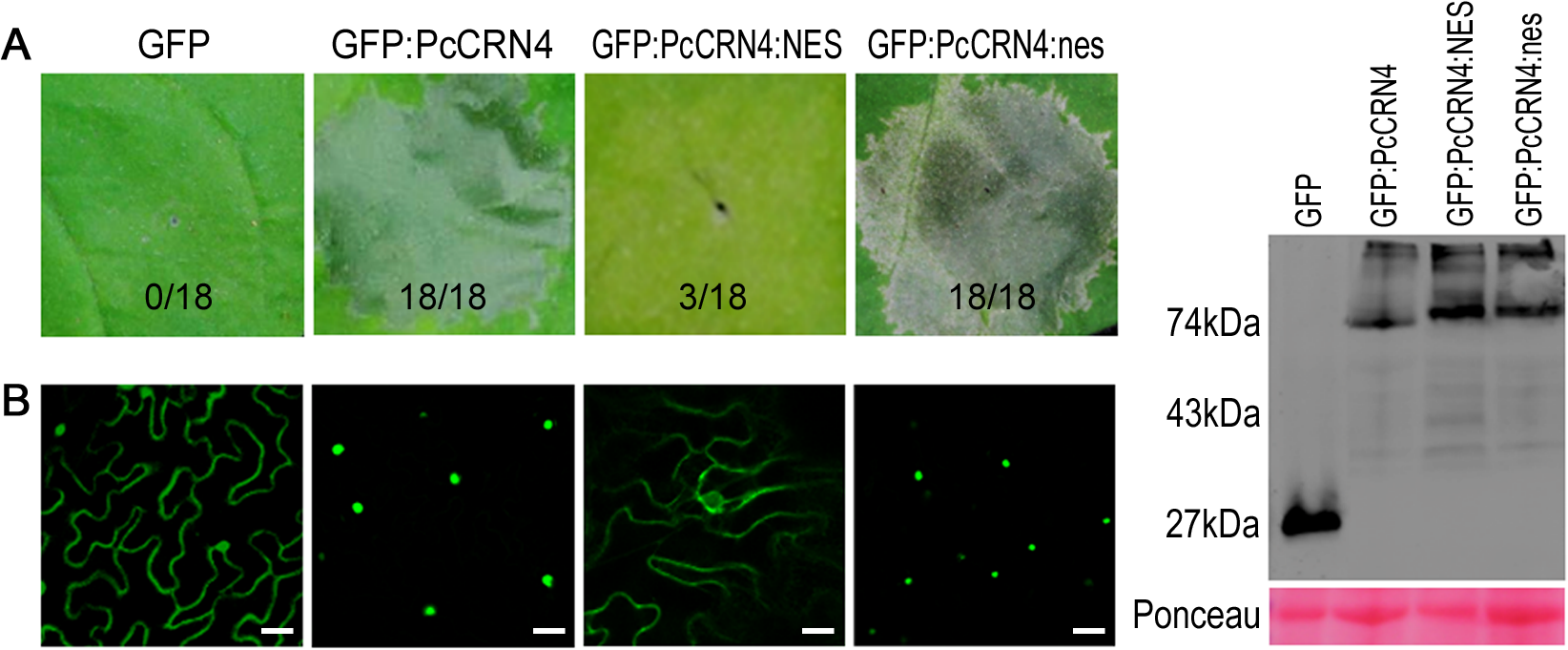
PcCRN4 effector domains function in plant nucleus. (A) Nuclear localization is required for PcCRN4-inducing cell death. *N*. *benthamiana* leaves were infiltrated with *Agrobacterium* strains carrying the indicated constructs. The representative pictures were taken at 5 dpi. The number shows the cell death sites and the total infiltrated leaves for each gene. NES and nes represent the nuclear export signal and nonfunctional NES. (B) NES impairs accumulation of GFP:PcCRN4 in *N*. *benthamiana* nucleus. *N*. *benthamiana* leaves were agroinfiltrated with the indicated constructs 48 hours before assessment of GFP confocal imaging. Scale bars, 25 μm. (C) Immunoblot analyses of GFP fusion protein accumulation *in planta*. Total proteins were extracted at 48 hpi. Blots were probed with α-GFP antibody. Sizes in kDa are indicated on the left.

Previous reports have shown that PiCRN8 [22] and PsCRN63 [18] target the plant nucleus to trigger cell death. To determine whether PcCRN4 also targets the nucleus, we searched the potential nuclear localization signal (NLS) using cNLS Mapper [32] and found that PcCRN4 contained a predicted NLS amino acid region at 363–388 (LAEPVKRRKLNQMLPFEPVKRRKLNQ). We hypothesized that the nuclear localization is required for CD induction and then tested this by fusing a nuclear exclusion signal (NES) [33] to the C terminus of PcCRN4. The fused protein was ectopically expressed by agroinfiltration. PcCRN4:NES consistently failed to elicit cell death 5 dpi (Fig 3A). To exclude the possibility that the NES may interfere with the activity, its mutated counterpart (nes) was fused to the C terminus of PcCRN4. In the nonfunctional nes, the second and third Leu residues and the first Ile residue were all substituted with Ala residues [33]. This construct robustly induced cell death as the wild-type (Fig 3A). NES constructs prevented nuclear accumulation of PcCRN4 inferred from the localization experiments, whereas PcCRN4 fused to the mutated NES domain retained in the nucleus (Fig 3B). We used Western blot analysis to confirm that these protein fusions were correctly expressed and the resultant proteins were largely stable in plant cells as only low levels of free GFP was observed (Fig 3C). Thus, we inferred that PcCRN4 requires nuclear accumulation to induce plant cell death.

### *PcCRN4* enhances *Phytophthora* virulence on *N*. *benthamiana* and decreases ROS accumulation

**Fig 4:**
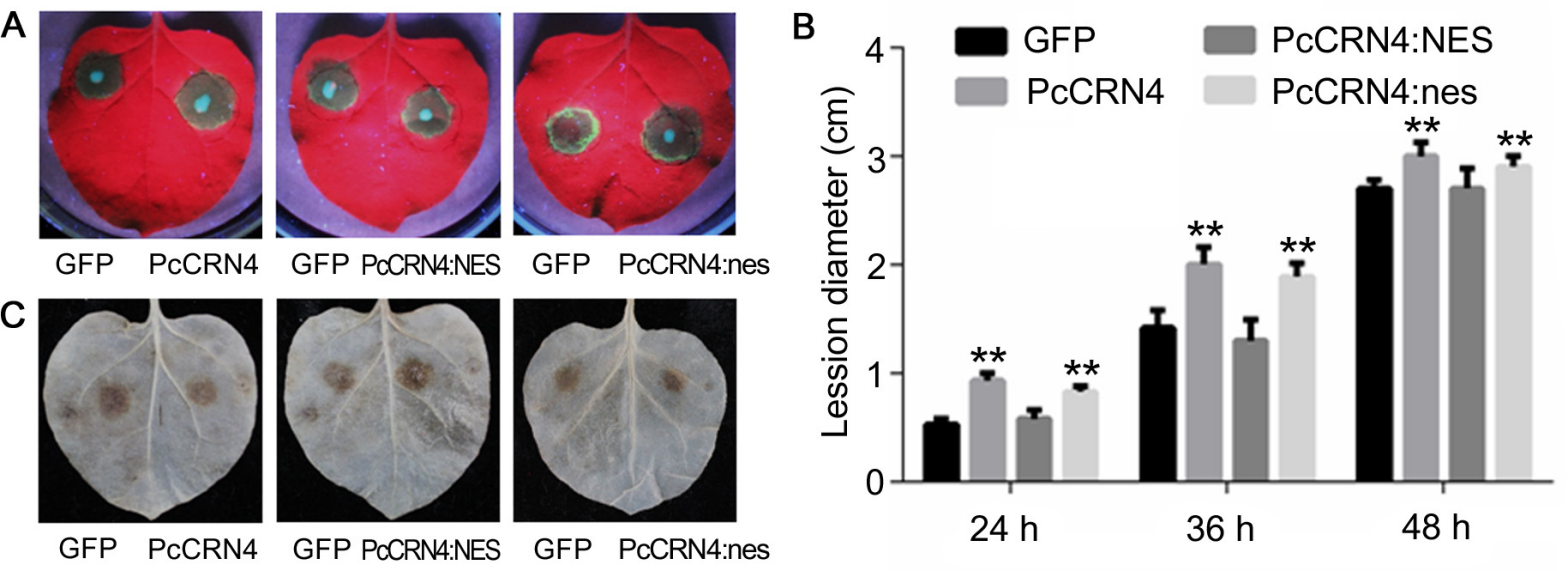
Expression of *PcCRN4* in *N*. *benthamiana* increased susceptibility to *Phytophthora* and suppressed ROS accumulation. (A) Observed phenotypes and (B) lesion diameters of *N*. *benthamiana* leaves inoculated with *P*. *capsici*. Ten μl (100 μl^-1^) zoospores were inoculated in the infiltrated regions 24 hours post infiltrated with the indicated genes and the photograph was then taken at 36 hours post inoculation. The lesion diameters were scored at the indicated time points. Statistical analyses were performed using a Dunnett’s test. (**, P < 0.01). (C) DAB staining of the inoculated sites of *N*. *benthamiana*.

Many oomycete effectors can suppress plant immunity responses [12,34–36]. Thus, we determined whether PcCRN4 interferes with plant immunity using plant transformation experiments by *Agrobacterium tumefaciens*-mediated transient expression of *PcCRN4*, *PcCRN4*:*NES* and *PcCRN4*:*nes* in *N*. *benthamiana*, whereas *GFP* was used as a control. Following agroinfiltration with the constructs (24 h later), we inoculated the infiltrated regions with *P*. *capsici* zoospores (10 x 100 zoospores μl^-1^) and evaluated disease development at the indicated times following inoculation (Fig 4A). On leaves infiltrated with strains carrying control gene (*GFP*), the diameter of the diseased lesion was approximately 1.2–1.5 cm at 36 hpi, which was similar to *PcCRN4*:*NES* at 1.3–1.7 cm; however, the lesion diameter expanded to 1.9–2.5 cm and 1.8–2.4 cm on leaves with strains carrying *PcCRN4* and *PcCRN4*:*nes*, respectively (Fig 4B).

To explore mechanisms behind the increased *Phytophthora* susceptibility of PcCRN4, diaminobenzidine (DAB) was used to examine infected plant tissues for H_2_O_2_ production [28]. Less DAB staining was observed (12 hpi) in infected regions of PcCRN4 and PcCRN4:nes compared to the PcCRN4:NES and GFP control (Fig 4C), indicating that PcCRN4 needs to target the plant nucleus to suppress H_2_O_2_ accumulation. Taken together, we showed that PcCRN4 needs to target plant nucleus to suppress plant defenses and promote colonization of the pathogen.

### PcCRN4 is required for full virulence

**Fig 5:**
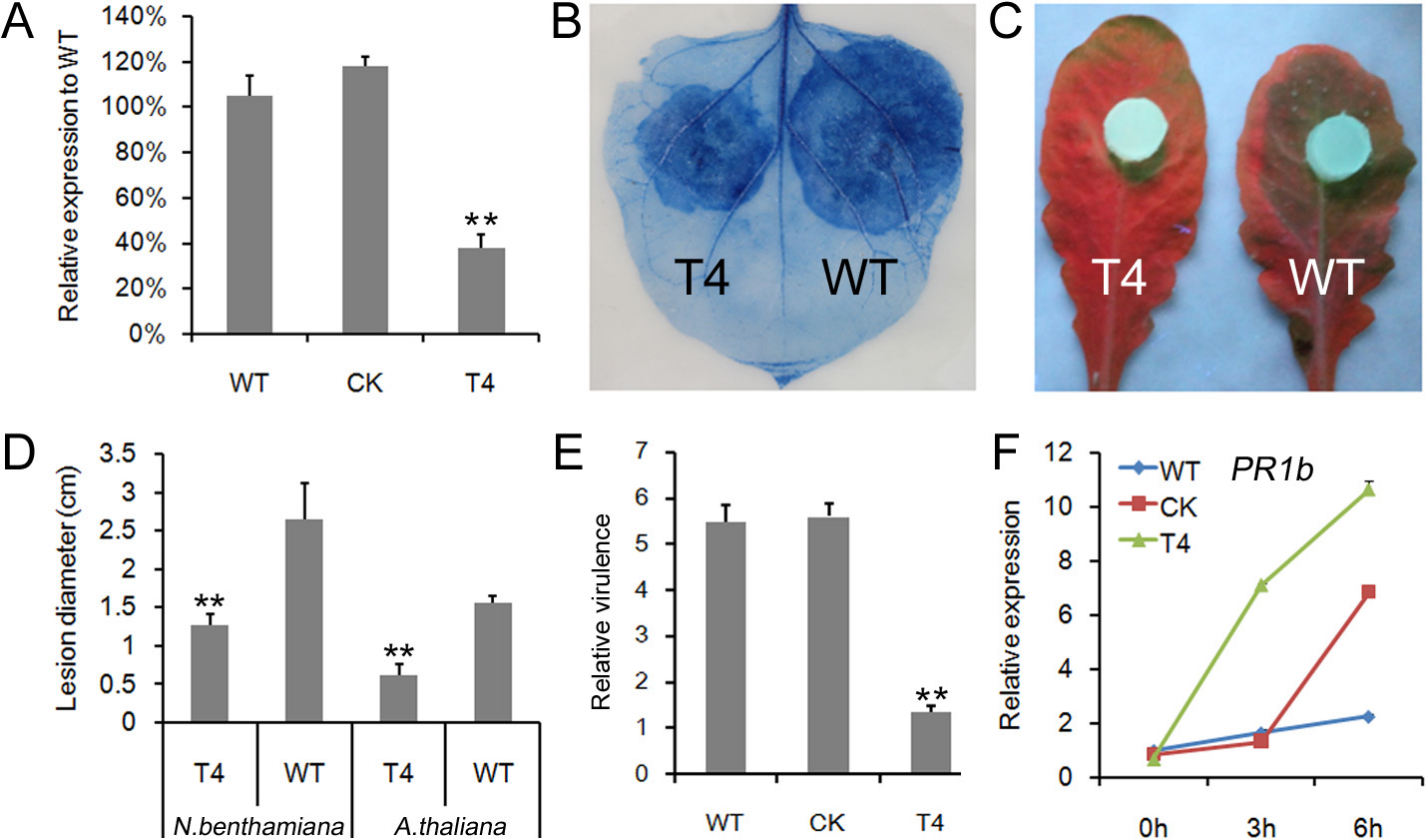
*PcCRN4*-silenced line showed reduced virulence. (A) Generation of a transformant with the silenced *PcCRN4* gene. Relative expression of *PcCRN4* in the silenced line (T4), unsilenced line (CK) and the wild type (WT) is shown. *P*. *capsici actin* transcripts were used as a reference and then normalized to the wild-type. Each bar represents the mean of three independent experiments with SE. (B and C) Lesions induced by the silenced line T4 and WT on *N*. *benthamiana* (B) and *A*. *thaliana* (C) leaves. The typical photos were taken at 36 hpi (B) and 30 hpi (C). (D) Lesion diameters of the inoculated sites. The data was measured at 36 hpi (*N*. *benthamiana*) and 30 hpi (*A*. *thaliana)* from over three independent replicates (**, P < 0.01, Student *t*-test). (E) Relative DNA amount in *P*. *capsici* in *N*. *benthamiana*. The relative virulence was calculated by Q-PCR assays of pathogen DNA levels in infected leaves relative to host DNA at12 hpi. Error bars represent SD from three technical replicates. (**, P < 0.01, Student *t*-test). (F) Relative expression of *PR1b* in *N*. *benthamiana* leaves. *P*. *capsici actin* gene was used as a reference and then normalized to the uninfected leaves (0 h) (**, P < 0.01, Dunnett’s test).

To directly test the contribution of PcCRN4 to *P*. *capsici* virulence, stable silencing of *PcCRN4* gene in *P*. *capsici* was carried out by polyethylene glycol (PEG)-mediated transformation method with the sense construct *PcCRN4* gene [18,26]. One silenced transformant (T4) was identified from 10 putative transformants that could grow on selective medium containing 50 μg ml^-1^ G418 and PCR screening. This transformant (T4) had the expression level of PcCRN4 reduced to <40% of the wild-type according to qPCR results (Fig 5A). The T4 transformant showed significantly reduced virulence on *N*. *benthamiana* at 36 hpi and *A*. *thaliana* at 24 hpi leaves as compared to the wild type (P<0.01) (Fig 5B–5D). To measure virulence of the transformant more precisely, we quantified the host and pathogen DNA by qPCR 12 h after inoculation [37,38]. The relative virulence was significantly reduced in T4 as compared to WT and another transformant in which *PcCRN4* gene was not silenced (Fig 5E). To investigate the effect PcCRN4 on host resistance, transcript accumulation of pathogenesis-related gene *PR1b* was examined. When the *N*. *benthamiana* leaves were inoculated with T4, rapid and increased levels of the transcriptions were observed at early stages of infection more than the non-silenced lines (Fig 5F). These results together with the virulence data indicated that PcCRN4 plays an important virulent role during infection.

**Fig 6:**
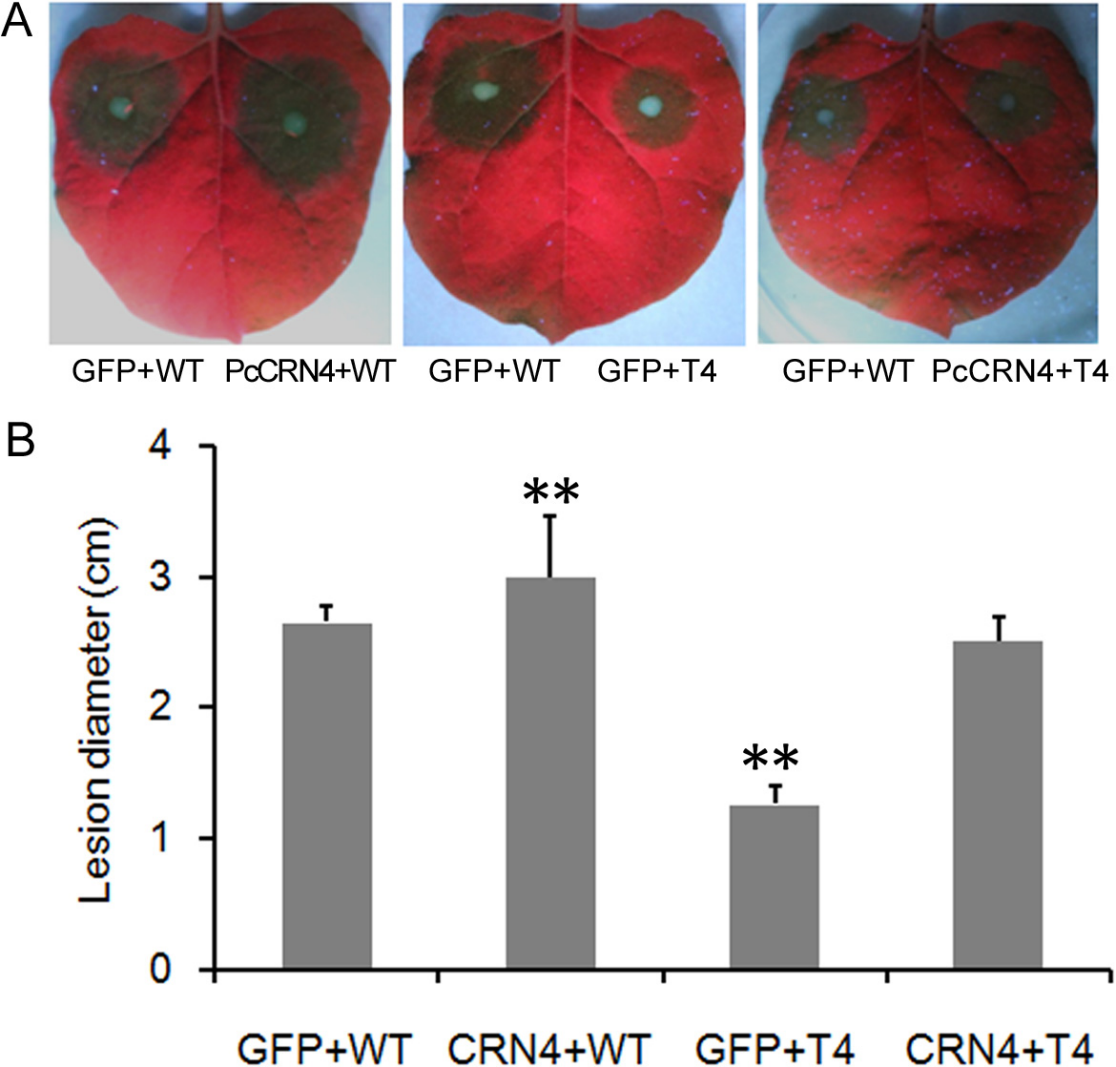
*In planta* expression of *PcCRN4* recovers virulence of the silenced line. Observed phenotypes (A) and lesion diameters (B) of *N*. *benthamiana* leaves. Ten μl (100 μl^-1^) zoospores of each line were inoculated in the infiltrated regions 24 hours post infiltrated with *PcCRN4* or *GFP* and the photographs were then taken at 36 hours post inoculation. The average lesion diameters were calculated from over three independent replicates. (**, P < 0.01, Dunnett’s test).

To determine the effect of PcCRN4 on T4 growth *in planta*, we infiltrated *N*. *benthamiana* leaves with *PcCRN4*, and 24 h later inoculated the leaves with T4 and WT zoospores. The lesion diameter of T4 increased from 1.3 cm when inoculated with *GFP* to 2.7 cm with *PcCRN4* at 36 hpi (Fig 6A and 6B). Collectively, the results suggested that *in planta* expression of *PcCRN4* can complement the *PcCRN4* silencing line based on the virulence tests.

**Fig 7:**
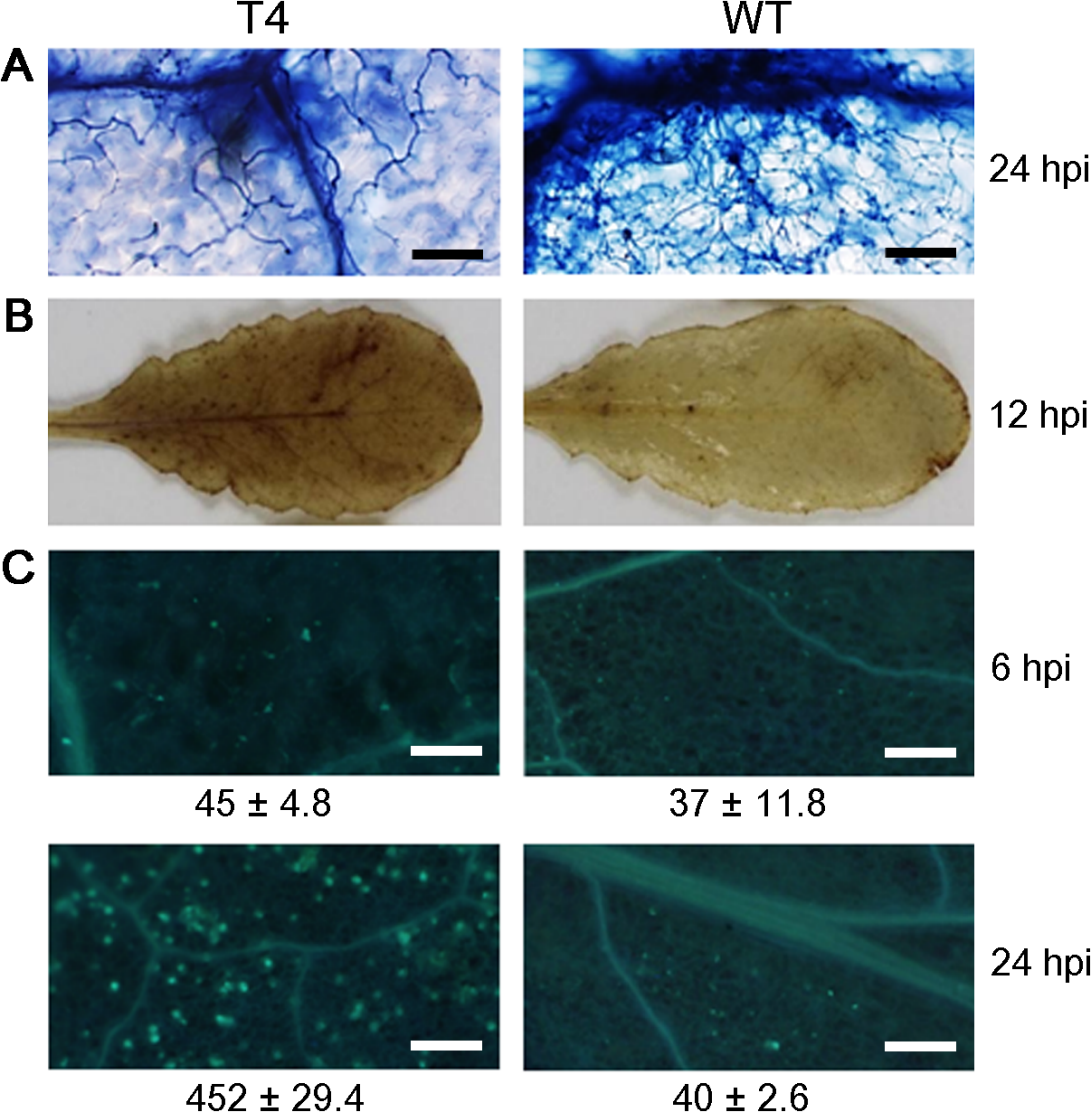
Silenced line could not suppress host H_2_O_2_ accumulation and callose deposition. (A) Trypan blue staining of the inoculated *A*. *thaliana* leaves. The typical photographs were taken after decolorizing with chloral hydrate 24 hpi. Bar, 40 μm. (B). Reactive oxygen species (ROS) accumulation. The inoculated leaves were stained with DAB stain and photographs were taken at 12 hpi. (C) Callose deposition detected with aniline blue staining. Data under the photos shows the relative callose intensities at 6 and 24 hpi from four replicates. The average number of callose deposits per microscopic field of 1 mm^2^ was calculated using the ImageJ software. Bars, 100 μm. T4, silenced line, WT, wild-type.

To analyze pathogen development in the host, we used an inverted microscope to visualize hyphae in infected tissue. At 24 hpi the transformant T4 and WT on infected *A*. *thaliana* leaves were stained with trypan blue. Abundant *P*. *capsici* hyphae were observed in tissues of WT but few hyphae were present in the tissues of silenced line T4 (Fig 7A). This result suggested that *PcCRN4* is required for full development of *P*. *capsici*.

DAB staining was performed on *A*. *thaliana* leaves, strong staining was observed in the silenced line T4 as compared to the WT at 12 hpi (Fig 7B). This result indicated that *PcCRN4* may promote *Phytophthora* infection by suppressing the H_2_O_2_ accumulation. Callose deposition was performed on the silenced line T4 and WT on *A*. *thaliana* leaves as described methods [29]. At 6 hpi, the callose deposits were almost undetectable and the same in T4 and WT. However, a strong signal of callose deposition was only found at 24 hpi in *A*. *thaliana* cells inoculated with WT (Fig 7C). This result indicated that a reduction in expression of the *PcCRN4* in *P*. *capsici* resulted in loss of abilities to suppress PTI in host cells.

### PcCRN4 may manipulate cell death by regulating expression of cell death-related genes

**Fig 8:**
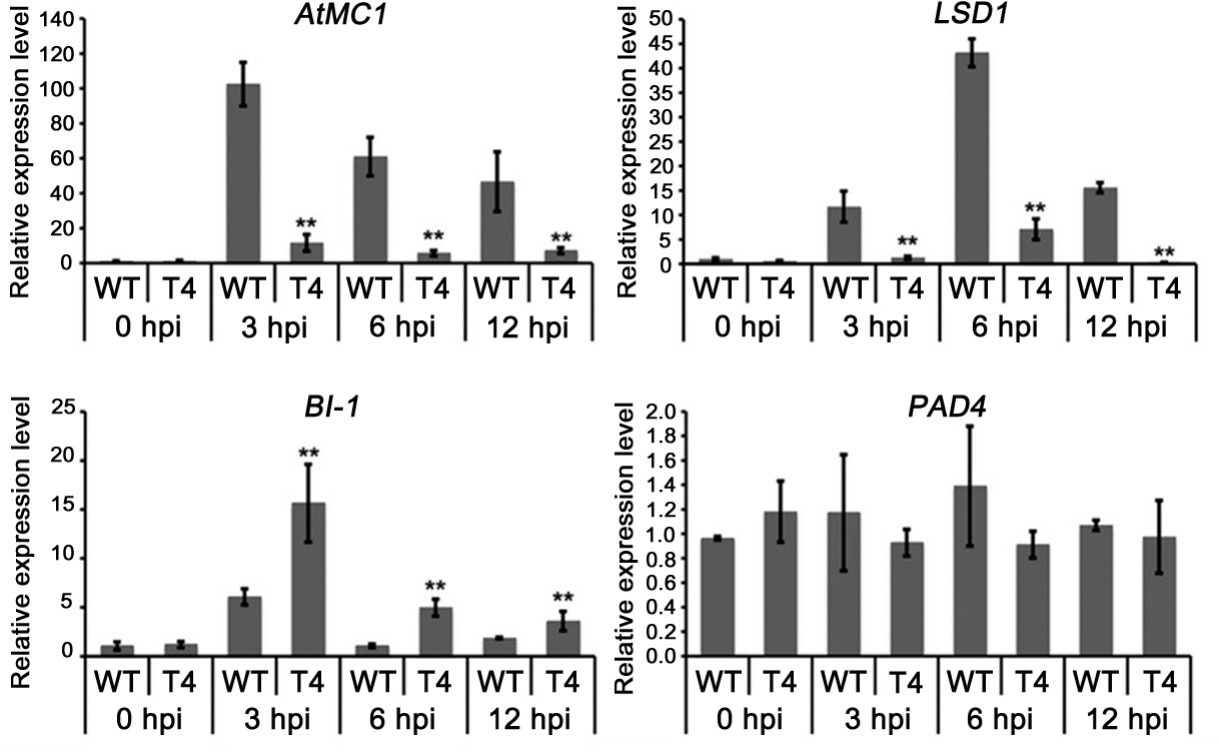
Relative expression levels of cell death-related genes in *Arabidopsis* after infection with wild-type and *PcCRN4*-silenced line. The relative levels of transcript were calculated by the comparative Ct method. Expression on leaves of *A*. *thaliana* inoculated with pathogen for 0 hr was fixed as one. Transcript levels of *UBQ5* gene of *Arabidopsis* were used to normalize different samples. Bars represent means and standard deviations of three replications. Asterisks indicate statistical differences between the transcripts of WT and T4 (P<0.01, Dunnett’s test).

Since PcCRN4 must enter plant cell nucleus to induce cell death, it is likely to affect the transcriptional levels of plant genes involved in cell death. Therefore, we tested expression levels of four well-known cell death-related genes (*AtMC1*, *AtLSD1*, *AtBI-1* and *AtPAD4*) in *Arabidopsis* after inoculation with wild-type and *PcCRN4*-silenced line. Transcripts of *AtMC1* and *AtLSD1* genes, which are positive regulators of cell death [39,40], were down-regulated in *Arabidopsis* leaves inoculated with *PcCRN4*-silenced line compared to that infected with wild-type (Fig 8). However, expression of another positive cell death regulator *AtPAD4* [41] was not significantly affected (Fig 8). These results suggested that PcCRN4 triggers cell death via a pathway dependent on AtMC1/AtLSD1 but independent on AtPAD4. Interestingly, transcripts of the broad-spectrum cell-death suppressor *AtBI-1* [41,42] were significantly higher in the *PcCRN4*-silenced line, compared to wild-type-infected plant tissues, suggesting that PcCRN4 promotes cell death partially by suppressing expression of the cell death inhibitor AtBI-1. Taken together, these results suggested that PcCRN4 promotes cell death by regulating expression of cell death-related genes.

## Discussion

Oomycete CRN proteins were initially identified through their ability to induce crinkling and necrosis when expressed in plant tissue, consequently this protein family is generally considered as a class of CD-triggering effectors [16]. However, recent studies suggested that the majority of CRNs could suppress CD triggered by PAMPs or other elicitors [15,19,23]. A recent study revealed that some CRN domains in *P*. *capsici* induce necrosis when expressed *in planta* [20]. Our study showed that PcCRN4 induced cell death in *N*. *benthamiana*, *N*. *tabacum* and *S*. *lycopersicum*, suggesting that PcCRN4 may target conserved and critical host proteins or signaling pathways.

We previously identified *P*. *sojae* effectors *PsCRN63* and *PsCRN115* and determined their expression patterns. *PsCRN63* was found to be induced during the late infection stages (12 and 24 hpi), whereas the highest levels of *PsCRN115* RNA were found at the mycelium stage [18]. We analyzed the expression pattern of the *PcCRN4* gene during infection. *PcCRN4* transcripts were low in the mycelium, motile zoospores and geminating cysts before infection. The transcripts accumulated during infection stage and reached maximum at 12 hpi, and their abundance dropped rapidly in late infection stages. Earlier studies on gene expression during pre-infection stages indicated that the greatest changes in *P*. *capsici* occurred during cyst germination compared with mycelium and zoospores [43]. Nevertheless, another study found evidence of a distinct biotrophic phase followed by a transition to necrotrophy after 24 hpi and sporulation at 72 hpi on susceptible tomato by *P*. *capsici* [44]. The gene expression pattern of *PcCRN4* suggests that it is involved in the initial manipulation of host cell death and defenses.

Emerging evidence has shown that oomycete and fungal pathogen effectors can enter inside plant cells to promote virulence [5,6]. Our results showed that PcCRN4 was required for the full virulence, and can induce cell death and enhance susceptibility when it was overexpressed in *N*. *benthamiana*. Its functions in plant cells are similar to its homologues effector PsCRN63 from *P*. *sojae* [18], but contrary to other effectors. For example, other cell-death-inducing CRN effectors could not enhance virulence [20]. Plant cell death is not only a major consequence of pathogen infection, but also prevent pathogen growth [39]. *P*. *capsici*, as a hemibiotrophic and wide-host-range pathogen, is assumed to evade host CD during the biotrophic phase but promote it during the necrotrophic phase. Since this gene is mostly expressed at the biotrophic phase, it is still unclear how CD-inducing activity of PcCRN4 contributes to its virulence. Considering that PcCRN4 may suppress plant ROS accumulation, *PR1b* gene expression and callose deposits, we speculate that this effector can suppress plant immunity when other effectors that can block PcCRN4-induced-CD are present. This is consistent with that two *P*. *sojae* RXLR effectors (Avh172 and Avh238) that can regulate host CD and play a positive role in infection [38].

Many *Phytophthora* CRN proteins are nuclear effectors [15,18,20]. We demonstrated that PcCRN4 need plant nuclear localization for its virulent and CD-inducing activities, although its biochemical mechanisms are unknown. The nucleus is the heart of plant cells and is also important for plant immunity. It has been shown that some bacterial effectors enter the host nucleus to interfere with transcription, chromatin-remodelling, RNA splicing or DNA replication and repair. These effectors are called ‘nucleomodulins’ or ‘nuclear attacks’ [45]. Thus, PcCRN4 belongs to a group of oomycete ‘nucleomodulins’ which might have permanent genetic or epigenetic effects on the hosts. Our results suggest that PcCRN4 promotes cell death by regulating transcription of cell death-related genes. However, it is still unknown whether PcCRN4 regulates expression of these cell death-related genes by direct binding to their promoter regions to promote/suppress transcription. Future identification of its host targets, including DNA and nuclear proteins, might lead to a better understanding or its functions in the nucleus.

## Supporting Information

S1 Fig Phenotypic analyses of CRN effector domains *in planta*. Four CRN effectors induced cell death in *Nicotiana benthamiana* and two induced death in both *N*. *benthamiana* and *N*. *tabacum*. The rest of the CRN effectors could not induce cell death. The experiment was repeated three times each with four infection sites per construct. The photos were taken at 5 dpi for *N*. *benthamiana* and 8 dpi for *N*. *tabacum*.
(TIF)

S1 Table Primers used in this study. (DOC)

S2 Table Summary of the screened CRN effectors. (DOC)
