# Supplementary figures and images for: A Virulence Essential CRN Effector of Phytophthora capsici Suppresses Host Defense and Induces Cell Death in Plant Nucleus
Source: PLoS One. 2015 May 26;10(5):e0127965. doi: 10.1371/journal.pone.0127965 (PMC4444017; doi:10.1371/journal.pone.0127965)

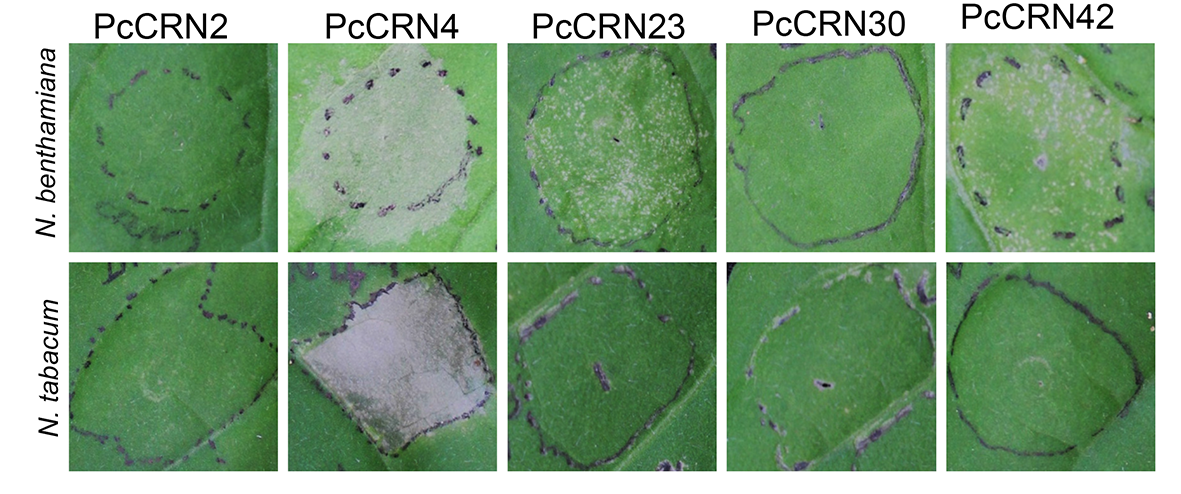

Supplement: S1 Fig — Four CRN effectors induced cell death in Nicotiana benthamiana and two induced death in both N. benthamiana and N. tabacum. The rest of the CRN effectors could not induce cell death. The experiment was repeated three times each with four infection sites per construct. The photos were taken at 5 dpi for N. benthamiana and 8 dpi for N. tabacum. (TIF) [file pone.0127965.s001.tif]
