# Supplementary material for: A Virulence Essential CRN Effector of Phytophthora capsici Suppresses Host Defense and Induces Cell Death in Plant Nucleus
Source: PLoS One. 2015 May 26;10(5):e0127965. doi: 10.1371/journal.pone.0127965 (PMC4444017; doi:10.1371/journal.pone.0127965)
Supplement: S1 Table — (DOC) [file pone.0127965.s002.doc]

**Table S1.** Primers used in this study

| **Primer** | **Sequence (from 5’ to 3’)** | **Purpose** |
| --- | --- | --- |
| pBINGFP2-CRN4-F | gggATGGAAATCGACGACAGCGC | To construct GFP:CRN4 for *in planta* expression |
| pBINGFP2-CRN4-R | ctagtctagaTTAACTGAAATAACTCAACGTGG |
| pBINGFP2-CRN4-NES-F | gggATGGAAATCGACGACAGCGC | To construct GFP:CRN4:NES for *in planta* expression |
| pBINGFP2**-**CRN4**-**NES**-**R | ctagtctagaTTACTACTTGTTAATATCAAGTCCAGCCAACTTAAGAGCAAGCTCGTTACTGAAATAACTCAACGTGGG |
| pBINGFP2-CRN4-nes-F | gggATGGAAATCGACGACAGCGC | To construct GFP:CRN4:nes for *in planta* expression |
| pBINGFP2-CRN4-nes-R | ctagtctagaCTACTTGTTAGCATCTGCTCCAGCTGCCTTAAGAGCAAGCTCGTTACTGAAATAACTCAACGTGGG |
| pTOR-CRN4-F | accggaattcAAAACCCAGCGACGATTA | To construct pTOR:CRN4 for gene silencing |
| pTOR-CRN4-R | ctagtctagaGACAAAGCCTGGAACTCG |
| CRN4-F | GTTTCCGCATTACTTACCC | qRT-PCR |
| CRN4-R | TGCTGATTTCACCTCCCT |
| Pcactin-F | GTACTGCAACATCGTGCTGTCC | qRT-PCR |
| Pcactin-R | TTAGAAGCACTTGCGGTGCACG |
| NbEF1a-F | AGAGGCCCTCAGACAAAC | qRT-PCR |
| NbEF1a-R | TAGGTCCAAAGGTCACAA |
| PR1b-F | GTGGACACTATACTCAGGTG | qRT-PCR |
| PR1b-R | TCCAACTTGGAATCAAAGGG |
| UBQ5-F | GGTGCTAAGAAGAGGAAGAAT | qRT-PCR |
| UBQ5-R | CTCCTTCTTCTGGTAAACGT |
| AtMC1-F | TATCCCGACCAAGCAAAA | qRT-PCR |
| AtMC1-R | TAGCCATCAACTTCATCACC |
| AtLSD1-F | TGTCAAACTACGAACCTTGTGC | qRT-PCR |
| AtLSD1-R | TTGATCTGCGCAACCTGA |
| AtBI-1-F | GCAGCAGCAATGTTAGCAAG | qRT-PCR |
| AtBI-1-R | CACCACCATGTATCCCACAA |
| AtPAD4-F | GGTTCTGTTCGTCTGATGTTT | qRT-PCR |
| AtPAD4-R | GTTCCTCGGTGTTTTGAGTT |

Notes: Restriction sites introduced into the amplicons are underlined.
