# Supplementary material for: A Virulence Essential CRN Effector of Phytophthora capsici Suppresses Host Defense and Induces Cell Death in Plant Nucleus
Source: PLoS One. 2015 May 26;10(5):e0127965. doi: 10.1371/journal.pone.0127965 (PMC4444017; doi:10.1371/journal.pone.0127965)
Supplement: S2 Table — (DOC) [file pone.0127965.s003.doc]

**Table S2. Summary of the screened CRN effectors**

| **Effector** | **Cell death** | | **RNA -seq** |
| --- | --- | --- | --- |
|  | *N. benthamiana* | *N. tabacum* | **RPKM value** |
| PcCRN1 | - | - | 280.012 |
| PcCRN2 | - | - | 136.612 |
| PcCRN3 | - | - | 170.663 |
| PcCRN4 | +++ | +++ | 94.0137 |
| PcCRN5 | - | - | 89.0416 |
| PcCRN6 | - | - | 82.9823 |
| PcCRN7 | - | - | 92.4728 |
| PcCRN8 | - | - | 64.8806 |
| PcCRN9 | - | - | 45.06 |
| PcCRN10 | - | - | 38.1915 |
| PcCRN12 | - | - | 31.4748 |
| PcCRN13 | - | - | 34.044 |
| PcCRN14 | - | - | 25.0753 |
| PcCRN15 | - | - | 17.9524 |
| PcCRN18 | - | - | 23.4632 |
| PcCRN20 | - | - | 26.3026 |
| PcCRN21 | - | - | 11.3961 |
| PcCRN22 | - | - | 11.3961 |
| PcCRN23 | + | - | 19.7395 |
| PcCRN24 | - | - | 14.7183 |
| PcCRN25 | - | - | 8.73145 |
| PcCRN26 | - | - | 12.8394 |
| PcCRN27 | - | - | 9.53993 |
| PcCRN28 | - | - | 8.38897 |
| PcCRN29 | - | - | 7.95122 |
| PcCRN30 | - | - | 8.39718 |
| PcCRN31 | - | - | 8.55087 |
| PcCRN33 | - | - | 4.69448 |
| PcCRN35 | - | - | 12.2838 |
| PcCRN37 | - | - | 4.91001 |
| PcCRN39 | - | - | 11.3905 |
| PcCRN40 | - | - | 6.74738 |
| PcCRN41 | - | - | 13.2535 |
| PcCRN42 | + | - | 13.2535 |
| PcCRN45 | - | - | 3.31592 |
| PcCRN46 | - | - | 7.0575 |
| PcCRN47 | - | - | 3.45449 |
| PcCRN48 | - | - | 3.45449 |
| PcCRN49 | - | - | 9.02945 |
| PcCRN52 | - | - | 4.13527 |
| PcCRN53 | - | - | 4.29287 |
| PcCRN54 | - | - | 4.57171 |
| PcCRN55 | - | - | 3.50074 |
| PcCRN56 | - | - | 2.71486 |
| PcCRN57 | - | - | 2.59196 |
| PcCRN58 | - | - | 1.38387 |

Notes: -, No cell death +, Weak cell death, +++, Strong cell death
